# Genome-Wide Identification of PIFs in Grapes (*Vitis vinifera* L.) and Their Transcriptional Analysis under Lighting/Shading Conditions

**DOI:** 10.3390/genes9090451

**Published:** 2018-09-07

**Authors:** Kekun Zhang, Ting Zheng, Xudong Zhu, Songtao Jiu, Zhongjie Liu, Le Guan, Haifeng Jia, Jinggui Fang

**Affiliations:** College of Horticulture, Nanjing Agricultural University, Nanjing 210095, Jiangsu, China; zhangkekun1990@163.com (K.Z.); 2016204006@njau.edu.cn (T.Z.); 2014204002@njau.edu.cn (X.Z.); 2015204003@njau.edu.cn (S.J.); 2017204011@njau.edu.cn (Z.L.); 2017104011@njau.edu.cn (L.G.); 2014002@njau.edu.cn (H.J.)

**Keywords:** PIFs, grapes, phylogenetic analysis, gene expression, lighting and shading conditions

## Abstract

Phytochrome-interacting factors (PIFs), as the basic helix–loop–helix (bHLH) transcription factors, are the primary signaling partners for phytochromes (PHY) that play a key role in PHY-mediated light signal transduction. At present, there are few studies on PIFs in fruit trees. In order to clarify the status of PIFs in grapevines, we identified members of the grape PIFs family and conducted phylogenetic and expression analysis. We identified PIF1, PIF3, PIF4, and PIF7 in PIFs families of the grapevine (*Vitis vinifera* L.), which were distributed on four different chromosomes with similar gene structures. Except for the closer relationship with PIF1 of citrus, PIFs of grape were distant from the other fruit species such as apple, pear, peach, and strawberry. The *VvPIFs* (except *VvPIF4*) were located in the syntenic block with those from *Arabidopsis* *thaliana*, *Solanum lycopersicum*, or *Citrus sinensis.* In addition to PIF1, all PIFs in grapevines have conserved active PHYB binding (APB) sequences. VvPIF1 has a conserved PIF1-specific active PHYA binding (APA) sequence, while amino acid mutations occurred in the specific APA sequence in VvPIF3. Interestingly, two specific motifs were found in the PIF4 amino acid sequence. The photoreceptor-related elements in the *VvPIFs* promoter region were the most abundant. PIF1, LONG HYPOCOTYL 5 (HY5) and PIF3, PIF4, GIBBERELLIC ACID INSENSITIVE 1 (GAI1) may interact with each other and participate together in light signal transduction. The relative expression levels of the *VvPIFs* showed diverse patterns in the various organs at different developmental stages, of which *PIF4* was most highly expressed. Prior to maturation, the expression of *PIF4* and *PIF7* in the skin of the different cultivars increased, while the expression of all *PIFs* in the flesh decreased. The transcription level of *PIFs* in grape leaves was sensitive to changes in lighting and shading. Shading treatment was beneficial for enhancing the transcription level of *VvPIFs*, but the effect on *VvPIF3* and *VvPIF4* was time-controlled. We concluded that PIFs in grapevines are both conservative and species-specific. The identification and analysis of grape PIFs could provide a theoretical foundation for the further construction of grape light regulation networks.

## 1. Introduction

Plants undergo the developmental program of photomorphogenesis in the presence of light [1]. Phytochromes, acting as red/far red-light sensors, are one of the most important photoreceptors in plants, and have been investigated extensively in terms of photochemistry, protein structures, and transduction of light signal [2,3]. There are various types of phytochromes in plants. *PHYA*, *PHYB*, *PHYC*, *PHYD*, and *PHYE* were reported in *Arabidopsis thaliana*, *PHYA*, *PHYB*, *PHYC* in *Oryza sativa*, *PHYA1*, *PHYB1*, *PHYC1* and their homologous genes *PHYA2*, *PHYB2*, *PHYC2* in *Zea mays* [1]. PHYs exist in both P_r_ and P_fr_ forms. Red-light activates PHYs by converting the P_r_ form to the P_fr_ form, whereas far red-light inactivates P_fr_ by converting it back to the P_r_ form.

Phytochrome-interacting factors (PIFs), the primary signaling partners for phytochromes, are basic helix–loop–helix (bHLH) transcription factors that play a key role in PHY-mediated light signal transduction. Similar to phytochromes, PIFs have been discovered in a variety of plant lineages from bryophytes to angiosperms [4,5]. In various plants, there are many endogenous (gibberellins, auxin, ethylene), environmental (blue light, high temperature) and developmental signaling pathways (circadian clock, cell fate) intersecting with PIF activity in regulating various morphogenic responses [6].

In *Arabidopsis*, many PIFs have been identified and functionally characterized. PIF1 has been shown to play a major role in inhibiting light-dependent seed germination [7], and regulates key genes involved in chlorophyll biosynthesis to optimize the greening process in direct or indirect ways [8]. PIF3 could promote hypocotyl elongation in response to ethylene and the biosynthesis of chlorophyll and anthocyanin accumulation under light conditions [9], negatively regulates seedling de-etiolation along with other PIFs [10,11], and recently, PIF3 was also shown to modulate the freezing tolerance as a negative regulator of the expression of C-repeat binding factor (*CBF*) genes [12]. PIF4 regulates the stomatal development in response to different light qualities, chlorophyll degradation and leaf senescence in darkness, and the freezing tolerance of the plant [13,14,15]. Additionally, it can uniquely regulate hypocotyl elongation in response to light and diurnal conditions, and early flowering response to high temperature [16,17]. PIF5 negatively regulates the red-light-induced anthocyanin biosynthesis and participated in many pathways regulated by PIF4 [18]. PIF6 has two splice variants, a and b, of which the b-form participates in the regulation of seed dormancy [19]. PIF7, as an interactor of PHYB, regulates the shade avoidance responses by allowing its dephosphorylated form to bind to the auxin biosynthetic genes [20]. In the meanwhile, many PIFs could coordinate to modulate certain physiological responses. Leivar et al. [21] found that PIF7 could interact with PIF3 and PIF4 to maintain the low levels of PHYB, involved in the process of the seedlings de-etiolation under far red-light. PIF3 and PIF4 interact with BRASSINAZOLE-RESISTANT 1(BZR1) to regulate genes involved in the brassinosteroid signaling pathways by binding to the same G-box element [22,23]. PIF1 also negatively regulates both biosynthesis and accumulation of carotenoids coupled with PIF3 [24].

At present, PIFs have been widely studied in *Arabidopsis*, tomato, maize, rice and other herbs [1,25,26,27], but little in woody plants. Grape is an important fruit tree resource in the world. The members of the *PIFs* gene family in grapes have not yet been identified and their function in grapevine photomorphogenesis, fruit development, regulation of endogenous metabolism, and reaction to external environment remains to be studied.

In this study, we identified a total of four *PIF* genes in the grape using *AtPIFs* as a template. Afterwards, we carried out the gene structure analysis of the *PIFs* in the grape, other five herbaceous plants and three woody plants, and analyzed the amino acid conserved functional domains and the *cis*-elements in the promoters. To further clarify the function of those genes in grapes, we also studied the transcriptional levels of *PIFs* in different organs of grapevines, the sensitivity of *VvPIFs* to different stresses in the berries and leaves. The results of our study can provide theoretical support for the further construction of regulation network of PIFs in grape and other fruit trees.

## 2. Methods

### 2.1. Plant Materials and Treatments

#### 2.1.1. Grape Berries in Development

The 6-year-old grape varieties Summer black (SB) and Shine Muscat (SM) planted in the Jiangsu Academy of Agricultural Sciences were used as materials to study the transcription patterns of *PIFs* during grape berry development. Twenty vines of each variety were chosen and the berry samples (three biological replicates) were collected at the following four developmental stages (DS): (i) DS1, the early stage of fruit enlargement (20 days after anthesis); (ii) DS2, pre-*verasion* (10% of the berries began to change color); (iii) DS3, post-*verasion* (90% of the berries completed the color changing); and (iv) DS4, maturity (the content of total soluble solids (TSS) and titratable acid (TA) tended to be stable). Samples were randomly collected, and the location of vines and clusters were also considered. Young leaves, young tendrils, young stems, young roots were also sampled for tissue-specific expression analysis. Mature berries were separated into the skin and flesh, and then were immediately frozen in liquid nitrogen individually. All samples were stored at −80 °C for the subsequent analysis.

#### 2.1.2. Grape Leaves Used for Lighting/Shading Studies

Potted 2-year-old Summer Black grapes were used for the experiment. The grape seedlings grew in an artificial climate chamber at 25 °C overnight before the treatment. Tinfoil paper with no light transmittance was used as the light-shielding material, and the leaves at the 4th to 6th nodes were selected for testing. Half the blades are treated with light and half are shaded along the dividing line of the main vein. One control (normal illumination, Control Check (CK)) and two treatments were set in the test. One treatment was a shading treatment, and the other was a light treatment after 6 h of shading. The sampling time started from shading and was set at 0 h, 2 h, 4 h, 6 h, 8 h, 10 h, 12 h after shading. The collected leaves were immediately frozen in liquid nitrogen and stored at −80 °C.

#### 2.1.3. Grape Berry Samples Used for Lighting/Shading Studies

The 6-year-old grape varieties Summer Black in Wuhu, Anhui Province, China were tested. 20 bunches of grape berries were shaded with tinfoil paper and another 20 bunches without shading. Fruit bagging was performed 30 days after fruit setting, and samples were taken at 0 days (d), 15 d, and 30 d after bagging. The berry sample was collected and stored as described above.

### 2.2. Genome-Wide Identification and Annotation of Grape PIFs Genes

We downloaded the protein sequence of *Arabidopsis PIFs* from the TAIR Arabidopsis database [28] and then blasted the homologous sequence against the grape genome database. To obtain a precise list of grapevine *PIFs* genes, we searched and downloaded the annotated grapevine proteins from three public databases: the National Centre for Biotechnology Information (NCBI; http://www.ncbi.nlm.nih.gov/), the Genoscope [29] and the Grape Genome Database [30,31]. The choice of the candidate *PIF* was based on the E-value (1e^−5^) and the highest similarity scores. All the obtained sequences were stored in the InterProScan database [32] and the Conserved Domains Database [33] to confirm their completeness and existence of the core domains. Length of sequences, molecular weights and isoelectric points of deduced polypeptides were calculated by using tools provided at the ExPasy website [34,35]. The choice of candidate PIFs in *Brassica napus*, *Nicotiana tabacum*, *Solanum lycopersicum*, *Prunus persica*, *Malus domestica*, *Pyrus bretschneideri*, *Citrus sinensis*, *Fragaria ananassa* was also based on the E-value (1e^−5^) and the highest similarity scores to AtPIFs in NCBI.

### 2.3. Gene Structure, Phylogenetic, Conserved Motifs and Syntenic Analysis of PIFs Family

Exon and intron structures of the VvPIFs were determined based on their coding sequence (CDS) and the correspondent full-length gene sequences in NCBI. The gene structures were thereafter illustrated by the online program Gene Structure Display Server [36]. MEGA version 6 (Sudhir Kumar, Arizona State University, Temp, AZ, USA) was used to construct phylogenetic trees by the Maximum Likelihood (ML) methods and the bootstrap test carried out with 1000 replicates [37]. The conserved motifs were identified using the online MEME program (version 4.12.0) [38]. We set the motif number as 10 and chose motifs with E-values ≤ 1e^−30^.

MCscan X (Yupeng Wang, University of Georgia, Athens, GA, USA) was used to analyze the gene synteny and collinearity of PIFs among *Vitis vinifera*, *A. thaliana*, *S. lycopersicum*, and *C. sinensis* [39]. The synteny figures were drawn by Circos-0.69 [40] and those results with E-value > 1e^−5^ were filtered.

### 2.4. Multiple Sequence Alignments, Promoter Analysis and Interaction Protein Prediction

Multiple sequence alignments of *PIFs* were performed using the MEGA version 6 [37]. The 1500 bp upstream of the *PIFs* genes of each species was used to perform *cis*-elements analysis in PlantCARE [41]. The protein sequences of VvPIFs were downloaded and used for interaction analysis and prediction in the online software STRING (ELIXIR, Hinxton, UK, https://string-db.org).

### 2.5. Transcriptome and Microarray Data Acquisition and Analysis

The transcriptome data of organs such as roots, stems, leaves, flowers, berries, and tendrils are from Gene Expression Omnibus (GEO, available online: https://www.ncbi.nlm.nih.gov/geo/). Data code was GSE36128 and the chosen cultivar was Corvina. The short term abiotic stress data was from GSE31594, in which Cabernet Sauvignon was treated with 120 mM salt (10:1 NaCl:CaCl), Polyethylene Glycol, cold (5 °C) or unstressed. The long-term salt and water stress data was from GSE31677, where the potted Cabernet Sauvignon vines in the greenhouse were exposed to irrigated controls, non-irrigated water-deficits, and saline treatments for 16 days. The expression data were normalized according to Zhu et al. [31]. The transcriptome data for strawberry, citrus, and apple fruits during their developmental period were also downloaded from the GEO database and numbered GSE85572, GSE69432, and GSE64079.

### 2.6. Total RNA Isolation, cDNA Synthesis and Gene Expression Analysis

Total RNAs were isolated using a CTAB method according to Wang et al. [42]. Then the first strand cDNA was synthesized from 1 μg total RNA with P1 [an oligo(dT)_20_ primer], P02 (a random primer) and SuperscriptIII RNase H-RT kit from Invitrogen (Carlsbad, CA, USA) according to the manufacturer’s instruction. The cDNA was diluted at 1:10 for RT-PCR.

Real time-PCR (RT-PCR) was carried out using the CFX96 Real-Time PCR Detection system (Bio-Rad, Hercules, CA, USA). Reaction mixes volume was 10 μL, which included 5 μL of SYBR Green Supermix (Bio-Rad), 2 μL of diluted cDNA, and 0.2 μL of each primer. Each pair of quantitative real time PCR (qRT-PCR) primers were validated by cloning and sequencing of the RT-PCR product with this pair of primers. The efficiency of each primer pair was quantified using a PCR product serial dilution. All biological samples were assayed in technical triplicates. Ubiquitin and EF1γ were used as internal standards and for normalizing the expression. Expression levels were calculated based on the 2^−ΔCt^ method. Due to the low expression values of PIFs in some organs of the grape berries, expression levels in those cases were calculated based on the 2^−ΔΔCt^ method with the first stage sample chosen as a reference for convenience of observation.

### 2.7. Statistical Analysis

Statistical analysis was performed with the software SAS 9.2 (SAS Institute Inc., Cary, NC, USA). Differences between genotypes and tissues for a giving sampling date were analyzed by a two-way analysis of variance (ANOVA) followed by the Duncan’s multiple comparison test at *p* < 0.05. Correlation network analysis was conducted in the Heml1.0.3.7 software (Yu Xue, Huazhong University of Science and Technology, Wuhan, China) and the line chart was performed by Origin 2017 (OriginLab Corporation, Northampton, MA, USA).

## 3. Results

### 3.1. Identification of Grape PIFs

A total of 4 PIF members were identified in the three grape genome database [29,30,31]. The four members were named as *PIF1*, *PIF3*, *PIF4*, and *PIF7*, respectively and their corresponding serial numbers are LOC100264869, LOC100247781, LOC100262490, and LOC100262138 in NCBI, VIT_07s0005g05100, VIT_14s0060g00260, VIT_12s0028g01110, and VIT_17s0000g06930 in CRIBI, and GSVIVT00028516001, GSVIVT00031338001, GSVIVT00020814001, and GSVIVT00007914001 in the 12X grapevine genome database. The basic information of the four genes was listed in Table 1. It can be seen that the gene length of the four *PIFs* varied from 3617 to 5463 bp, and the length of amino acids sequence ranged from 423 to 709. *PIF3* and *PIF7* have the largest or the smallest genomic DNA, number of amino acids and protein molecular weight individually. The predicted isoelectric point range of various proteins ranged from 5.32 to 8.94, indicating that those proteins might function in different subcellular compartments. What is more, each protein contains a bHLH domain.

As for the comparison among CDS (Table 2), the similarity between each pair was more than 40%, with the highest similarity being between *PIF4* and *PIF1* (55%). But for the amino acid sequences, the similarity of each pair was low, ranging from 22% to 33%.

### 3.2. Phylogenetic, Conserved Structural and Syntenic Analysis of PIFs

**Figure 1:**
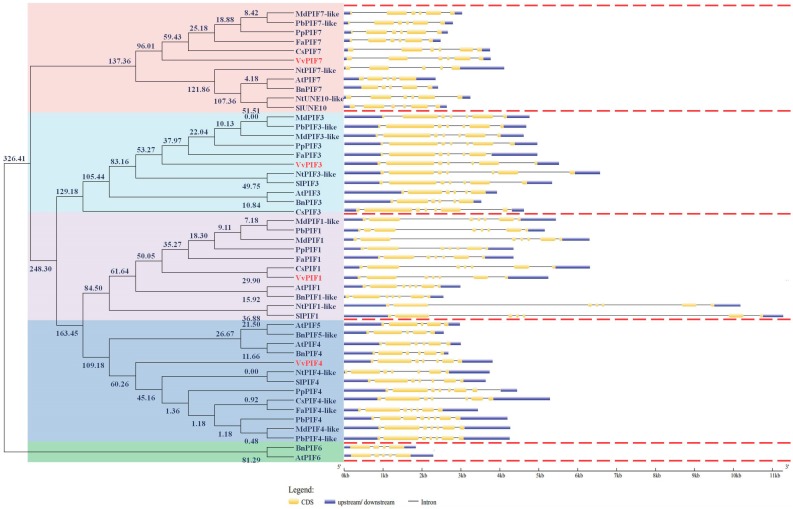
The phylogenetic tree of PIF proteins and their gene structure characteristics. Members of PIFs from the *Vitis vinifera* (red mark), *Arabidopsis thaliana*, *Brassica napus*, *Nicotiana tabacum*, *Solanum lycopersicum*, *Prunus persica*, *Malus domestica*, *Pyrus bretschneideri*, *Citrus sinensis*, and *Fragaria ananassa* were put together for comparison. The phylogenetic tree was generated by MEGA 6.0 using Maximum Likelihood method (timetree). The axis numbers mean the relative divergence time. CDS: Coding sequence.

According to the estimated divergence times for the molecular phylogenetic analysis by ML method (timetree) (Figure 1), the PIF family members were roughly divided into 5 categories (the relative divergence time at the branching points was larger than 150). PIF1 located in the first category, while PIF4 and PIF5 in the second, PIF3 in the third, PIF7 and UNE10 in the fourth, and PIF6 in the fifth. In the first category, PIF1 could be divided into two classes, SlPIF1 and NtPIF1-like were clustered together with a far kinship to the others. The fruit species were closely related, in which the kinship between the grape and the citrus was the closest. In the second category, the PIFs could also be broadly divided into two classes. PIF4, PIF5 in the *A. thaliana* and *B. napus* were clustered together and other species were clustered in another class. But in the second class, VvPIF4 had a far kinship with other fruit species. In the PIF3, CsPIF3 diverged from other species at the earliest time, and VvPIF3 had a distant relationship with other species. In the PIF7, VvPIF7 was at the starting node of fruit species classification and has a distant relationship with others. PIF6 was only found in *A. thaliana* and *B. napus*. Overall, except for the closer relationship with citrus on PIF1, PIFs of grapes were far from the other fruit species such as apples, pears, peaches, and strawberries.

From the gene structure of *PIF1* (Figure 1), it could be found that the *SlPIF1* was longest, *BnPIF1-like* was the shortest and the *PIF1* of fruit species was of moderate length. Except for *PbPIF1*, all plants contained seven exons in *PIF1*. In these exons, the second and the sixth exon were longer, others were shorter and the distance between exon3, 4 and 5 was short. For *PIF3*, the *NtPIF3-like* was the longest in sequence length while the *BnPIF3* was the shortest. There were seven exons in the *PIF3* of woody trees, of which the second and the sixth were longer. For *PIF4* and *PIF5* structure analysis, *BnPIF4* was the shortest and *CsPIF4* was the longest. There were seven exons in *VvPIF4*, with the similar structure to *PIF4* in other plants. As for *PIF7*, there were six exons in *VvPIF7*. After UTR end removed, *VvPIF7* was the longest. In general, the *PIF1* gene structure, with the stable number of exons and introns, was relatively conservative, but the gene length varied greatly between different species. The structure of *PIF3*, *PIF4*, and *PIF7* genes differed among different species.

**Figure 2:**
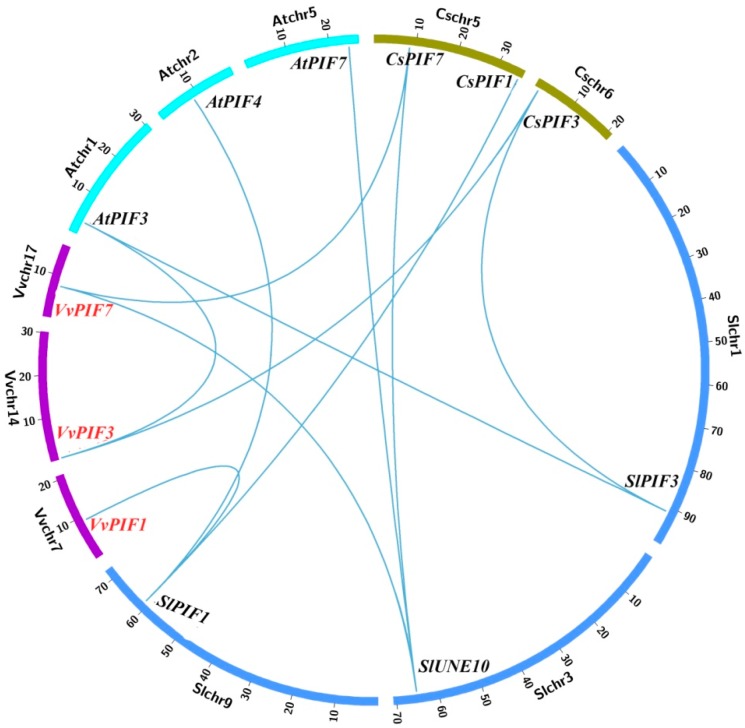
Syntenic block among *PIFs* from *V. vinifera*, *A. thaliana*, *S. lycopersicum*, and *C. sinensis*. The chromosomes of *V. vinifera*, *A. thaliana*, *S. lycopersicum*, and *C. sinensis* are displayed in purple, bright green, blue, gray green. The putative orthologous *PIFs* are connected by light blue.

According to the results of syntenic analysis (Figure 2, Table S1), we found that there were *VvPIFs* corresponding genes in *A. thaliana*, *S. lycopersicum*, and *C. sinensis*, and these genes were located at the same syntenic block. Combined with the relative evolution time analysis in Figure 1, *VvPIF7* evolved earlier, and might be derived from a common ancestor with *CsPIF7* and *SlUNE10*. *VvPIF3* evolved later than *CsPIF3*, and might had a common origin with *AtPIF3*. *VvPIF4* was not located in the syntenic block, and might had certain specificity due to its late evolution time. Arabidopsis, tomato and citrus are important model plants for studying herbaceous plants and fruit trees. The study of the collinear relationship of *PIFs* in these plants would shed important implications for the functional study of *VvPIFs*.

### 3.3. Comparison of Conserved Motifs

**Figure 3:**
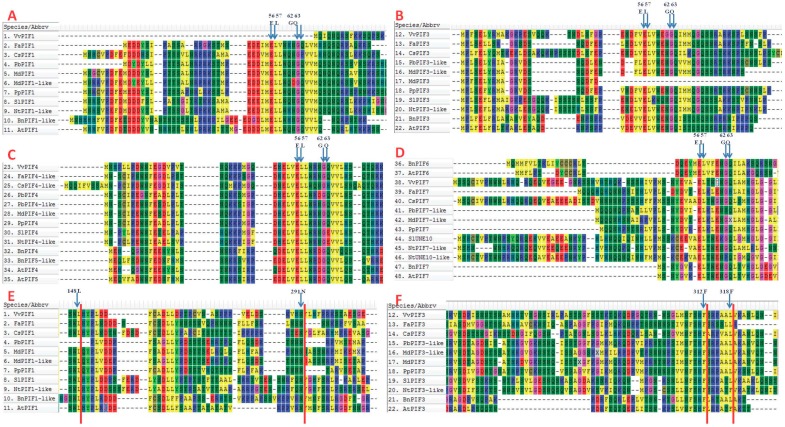
The multiple alignment of deduced amino acid sequences of PIFs for the comparison of active PHYB binding (APB) and active PHYA binding (APA) conserved domains. (**A**–**D**) Comparison of APB conserved domains in PIFs; (**E**) Comparison of APA conserved domains in PIF1; (**F**) Comparison of APA conserved domains in PIF3. The amino acid of the conserved site has been marked above the corresponding column.

The conserved motifs of active PHYB binding (APB) in *A. thaliana* were E41, L42, G47, and Q48 [43]; of PIF1- (active PHYA binding) (APA) were L95, N144, and of PIF3-APA were F203, F209 [44]. From the Figure 3, it could be found that the motifs of APB in VvPIF1, CsPIF1, MdPIF3, CsPIF4-like, SlPIF4, NtPIF4-like, FaPIF7, PbPIF7-like, MdPIF7-like, NtUNE10-like changed (Figure 3A–D), which might affect their combination with PHYB. The change in the APA conserved sequence of PbPIF1 indicated its poor binding to PHYA. In addition to AtPIF3 and NtPIF3, F209 of PIF3-APA in other plants all mutated to L. Since that F203 and F209 were mutated together in the previous motifs study [44], whether a single mutation occurs in these two sites affects the combination of PHYA with PIF3 remains to be further explored.

**Figure 4:**
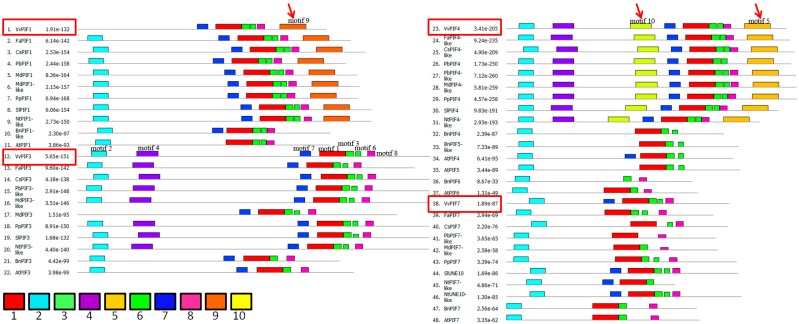
MEME analysis of the conserved motifs of PIFs protein. The motif number was set as 10 and one color corresponds to one motif. The amino acid sequence corresponding to each motif was present in Figure S1. The arrows indicate the specific motifs for PIF1 and PIF4 compared to other PIFs. The motif name of each color was labeled in VvPIF1, VvPIF3 and VvPIF4, respectively.

After conservative motifs analysis of PIFs family, we obtained 10 conserved domains, of which the second domain contained APB conserved motifs (Figure 4, Figure S1). A total of seven domains were detected in PIF1, and the fourth, fifth, and tenth motifs were missing. The conserved domains of AtPIF1 consisted of only five kinds. Grapes lack the second motif compared to other species, consistent with the results of the previous study (Figure 3). Except for grapes, motifs of other fruit species were similar. What’s more, motif 9 was a specific motif in PIF1 compared to other PIFs. Seven conserved motifs were detected in PIF3, these motifs were similar among fruit species, except MdPIF3. A total of 9 conserved motifs were detected in PIF4, and BnPIF4 had the least number with only 4 kinds of motifs. With the exception of PbPIF4, the motifs of other fruit species were similar. Compared with other motifs, motif 5 and motif 10 were specially detected in PIF4 and motif 4 in PIF3 and PIF4. In PIF7, six conserved sequences were detected in grapevines, similar to those in *S. lycopersicum* and *N. tabacum*, of which motif 1 and motif 2 existed in all fruit species, but others differed greatly among species. Above all, motif 1, 2, 7, 3, 6, 8 seem to be the conserved motifs of PIFs.

### 3.4. Cis-Element Analysis in the PIF Gene Promoters and Functional Prediction of PIFs Proteins

**Figure 5:**
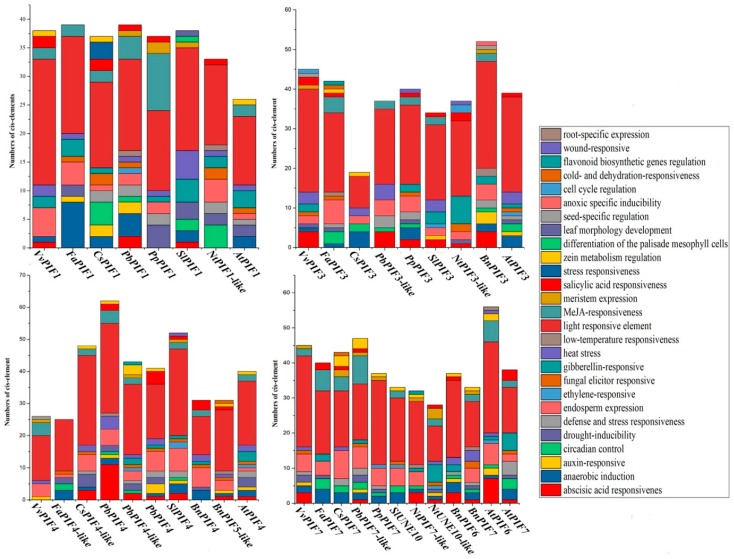
*Cis*-elements in the promoter of *PIFs* genes that are related to stress responses and plant development.

To further clarify the gene function and transcriptional regulation mechanism of *PIFs*, we amplified the 1500 bp upstream of the *PIFs* genes and analyzed their *cis*-elements. The predicted *cis*-elements differed among species and genes, but the *cis*-elements related to photoreaction were the most abundant (ACE, G-Box, GT1-motif, Sp1, ATCT-motif, Box 4, I-box, TCT-motif, AT1-motif), which had the largest number in all species (Figure 5).

The number of *cis*-elements in the promoters of PIFs was also various, and nine were detected in *VvPIF1*, 12 in *VvPIF3*, 7 in *VvPIF4*, and 11 in VvPIF7. In the promoter of *VvPIF1*, the number of endosperm expression related elements (Skn-1 motifs) was the largest except those related to photoreaction. In the promoter of *PIF3* and *PIF7*, there was many elements for abscisic acid responsiveness (ABRE), but no for circadian control (circadian: CANNNNATC) in grapes when compared with other fruit species. The *cis*-elements in the *VvPIF4* promoter were less diverse. Compared with other fruit trees, the upstream of *VvPIF4* lacked the anaerobic induction (ARE), drought-inducibility (MBS) related elements, and had a seed-specific regulation class (RY-Original) element. Last but not least, there were no low-temperature-responsive elements detected in the promoters of *VvPIFs*.

As we all know, PIFs are involved in the transduction of light signals in plants. However, the specific regulatory network of PIFs in grapes is still not clear. VvPIF1 could interact with PHYA, PHYE, and HY5 to participate in the regulation of diurnal growth and development of plants (Figure S2, Table S3). VvPIF3 not only combined with photoreceptors, but also interacted with DELLA protein GAI1 to participate in the signal transduction process of plants. PIF4 could also interact with GAI1 to regulate the growth process. In addition to the known phytochromes, hormone signaling-related proteins, etc., PIFs could also interact with many undefined functional proteins, and their metabolic network remained to be further studied. To further clarify the relationship between *PIF* gene and grape growth, we also performed expression analysis.

### 3.5. Expression Profiles of PIFs in Different Organs

**Figure 6:**
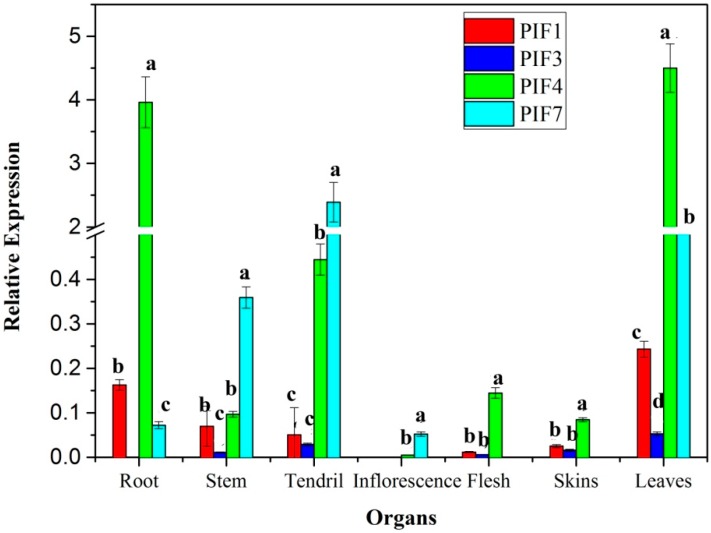
Relative expression levels of *Vinitus vinifera* phytochrome-interacting factors *(VvPIFs)* in different organs of Summer Black (SB). Values were normalized against the expression data of *VvUBI* gene and are given as means ± standard error among three biological replicates. Different letters indicate significant differences between genes (*p* < 0.05) according to Duncan’s multiple test. The expression levels were calculated based on the 2^−ΔCt^ method.

There are differences in the expression profiles of *PIFs* in grape organs (Figure 6). The transcriptional level of *VvPIF4* was the highest in roots, leaves, skin, and flesh, while *VvPIF7* showed the highest expression in stems, tendrils, and flowers. According to the Figure 6, *VvPIF1* and *VvPIF3* expressed higher in leaves than that in other tissues.

According to the transcriptome data of Corvina (Figure S3), the *VvPIF4* highly expressed in all tissues, except in the buds at germination stage (Bud-B, Bud-AB). *VvPIF3* was at higher transcriptional levels than *VvPIF4* and *VvPIF7* in most organs, such as the berries in stages before maturation (Berry Pericarp-FS, Berry Pericarp-PFS, Berry Pericarp-V), buds during sprouting and developing (Bud-S, Bud-B, Bud-L, Bud-W), the growing flower (Flower-FB, Flower-F), leaves before senescence (Leaf-Y, Leaf-FS), the developing rachis (Rachis-FS, Rachis-PFS), stems and tendril (Stem-G, Stem-W, Tendril-Y, Tendril-WD, Tendril-FS). Compared with the expression of other *PIFs*, *PIF7* was only expressed higher at Bud-B, Leaf-Y, Leaf-FS and Tendril, and the transcription level of PIF1 was low in most organs.

When comparing the transcriptional data of *PIFs* between SB and Corvina, the transcriptional levels of *PIF4* were similar and high in roots, mature leaves, and fruits (skin and flesh) of both varieties. But the relative level of *PIF7* was a little different in the young stem, tendril and inflorescence of these two cultivars, and the transcription level of *PIF7* in Corvina was not as high as that in SB. These differences may be related to the cultivar characteristic and developmental stage. Taken together, the transcription level of *PIF4* was the highest in most organs of the grape, followed by *PIF3* and *PIF7*.

### 3.6. Expression Profiles of PIFs During Grape Berries Development

**Figure 7:**
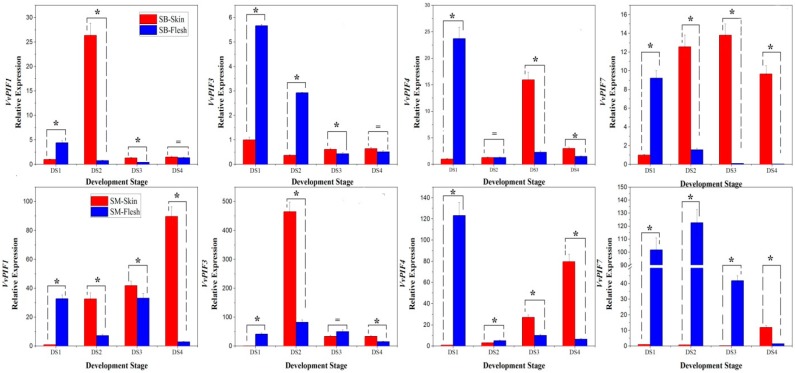
Relative expression levels of *VvPIFs* in the skin and flesh of SB (top panels) and Shine Muscat (SM; bottom panels) berries during different developmental stages (DS). Stars indicate significant differences between skin and flesh (*p* < 0.05) according to Duncan’s multiple test, while the equal sign means non-specific difference. DS1, DS2, DS3, DS4 represent the early stage of fruit enlargement, pre-*verasion*, post-*verasion* and maturity respectively. The skin of SB and SM were purple-black and yellow-green, respectively. Due to the low expression values of PIFs in these organs, expression levels were calculated based on the 2^−ΔΔCt^ method with the first stage sample chosen as a reference for convenience of observation.

The transcriptional level of *PIFs* during fruit development showed a different pattern in different colored grape varieties (Figure 7). In SB, the transcriptional levels of *PIF1* in grape berries slightly changed, except the short-term peaks appearing in the flesh during DS1 and in the skin during DS2. The transcriptional level of *PIF3* kept stable in the skin and decreased significantly in the flesh. The expression of *PIF4* and *PIF7* firstly increased and then decreased in the skin, whereas they showed overall downward trends in the flesh. In short, except the *PIF3* in the skin, the expression level of *PIFs* in SB showed a first increasing and then decreasing trend in the skin and an overall downward trend in the flesh. In SM, the expression of *PIF1* showed an overall increasing trend in skin and a fluctuating pattern in the flesh. The expression of *PIF3* showed a trend of first increasing and then decreasing both in the skin and flesh. The expression of *PIF4* and *PIF7* showed an overall upward trend in the skin and downward trend in the flesh. In short, except the *PIF3* in the skin, the expression level of *PIFs* in SM showed an increasing trend in the skin and an overall downward trend in the flesh. The expression patterns of *PIFs* in the flesh of the two grape varieties were similar, indicating that the coloration of the skin did not affect the rhythmic expression pattern of *PIFs* in the flesh.

### 3.7. Expression Profiles of PIFs under Different Treatments

**Figure 8:**
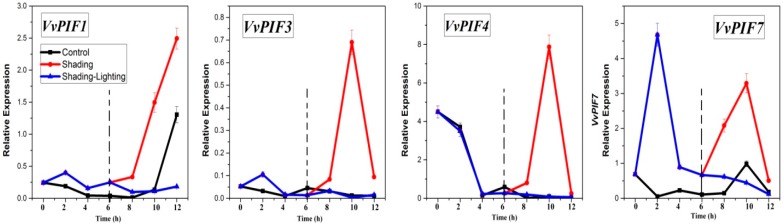
Relative expression levels of *VvPIFs* in the leaves of SB under light/shading conditions. The expression levels were calculated based on the 2^−ΔCt^ method.

The purpose of the lighting/shading experiment in leaves was to investigate the sensitivity of PIFs to light and dark conditions at the transcriptional level. The test was carried out in a thermostatic artificial climate chamber. A total of 12 h of light treatment, 12 h of dark treatment, and 12 h of light and dark alternate treatment were designed. Zero hours represented the starting point of the test, 12h represented the end of the test, and 6 h was especially important for shading-lighting treatment, representing the time of light and dark alternating. From 0 to 6 h, the expression of *PIF1* in grape leaves showed a decreasing trend under light conditions, and a fluctuating trend under shading conditions, with peaks appearing at 2 and 6 h (Figure 8). From 8 h to 12 h, the expression of *PIF1* increased both under light and shading, but changed slightly under shading-lighting conditions. Notably, the increasing trend under shading conditions was more obvious. These results indicated that dark treatment was beneficial to increase the expression level of *PIF1*, and sudden light treatment had an inhibitory effect on the intrinsic expression pattern of *PIF1*. Shading didn’t change the *PIF1* overall changing patterns. From 0 to 6h, the expression level of *PIF3* was close in shading and light conditions except for 2 h, However, at 10 h, the expression level of *PIF3* under dark conditions showed a clear peak, extremely higher than that under other treatments. The expression level of *PIF4* decreased from 0 to 6 h under shading and lighting conditions. Similar to the expression changes of *PIF3*, *PIF4* also showed a significant peak at 10 h. These results indicated that shading was beneficial to increase the expression of *PIF3* and *PIF4* in leaves, but the effect of shading was limited by circadian or the length of shading time. The expression level of *PIF7* was low and changed gently under light conditions from 0–6 h, while that showed a clear peak at 2 h and then decreased rapidly. From 6 to 12 h, the transcription level of *PIF7* under light treatment increased first and then decreased, while that under shading treatment showed a similar trend but changed more obviously. The expression level of *PIF7* in the shading-lighting treatment showed a continuous downward trend, indicating that sudden light conditions also had an inhibitory effect on the intrinsic expression pattern of *PIF7.* Leaf shading experiments showed that *VvPIFs* were sensitive to light changes. Shading treatment was beneficial to increase the expression level of *PIF1* and *PIF7*, but didn’t change its internal change pattern. Shading treatment also helped to increase the expression level of *PIF3* and *PIF4*, but the enhancement effect was time limited. In addition to *PIF3*, sudden light treatment after shading conditions inhibited the expression of *PIFs*.

**Figure 9:**
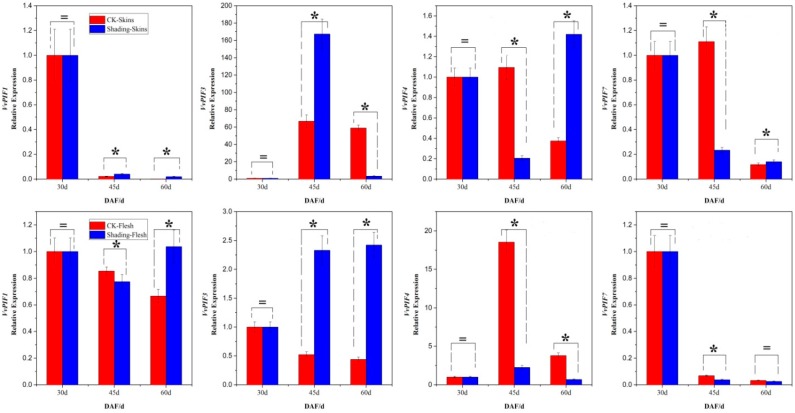
Relative expression levels of *VvPIFs* in the skin and flesh of SB under light/shading conditions. Stars indicate significant differences among different treatments (*p* < 0.05) according to Duncan’s multiple test, while the equal sign means non-specific difference. DAF indicates days after flowering. Due to the low expression values of PIFs in these organs, expression levels were calculated based on the 2^−ΔΔCt^ method with the first stage sample chosen as a reference for convenience of observation.

Fruit shading shed different effects on the expression patterns of *PIFs* in the skin and flesh (Figure 9). Compared with that in the control berries, the transcriptional level of *PIF1* of shading berries was higher both in the skin and flesh at maturity, which indicated that the shading treatment improved the expression levels of *PIF1* in grape berries at maturity. According to this analysis method, it could be found that the shading treatment improved the transcriptional level of *PIF3* in the skin only at 45d, while it promoted the level of PIF3 continuously in the flesh. Contrary to the change in transcription patterns of *PIF3*, the transcriptional level of *PIF4* in the skin was reduced at 45d and showed a constantly decreased trend in the flesh. After shading, the expression level of *PIF7* in the skin was improved at 60d, but showed no significant change in the flesh at that time. It was *PIF1*, *PIF3*, *PIF4*, and *PIF7* in the skin and *PIF1*, *PIF3*, and *PIF4* in the flesh that were sensitive to light/shading changes.

The effects of different abiotic stresses on the expression levels of *PIF4* and *PIF7* were different. Salt and polyethylene glycol (PEG) treatment did not change the trend of *PIF7* at different periods but increased the expression of *PIF7* at 1 and 24 h. The expression level of *PIF4* gradually increased under PEG treatment. Under low temperature conditions, the transcriptional levels of *PIF4* and *PIF7* were down-regulated, but the latter decreased more significantly (Figure S4C,D). Under the long-time abiotic stresses, salt treatment did not alter the expression levels of *PIF4* and *PIF7* at D4, D8, and D12, but significantly increased their expression level at D16, indicating that salt can increase the transcription level of *PIF4* and *PIF7*, without changing their developing patterns (Figure S4A,B). Under water deficit conditions, the transcription level of *PIF7* and *PIF4* was significantly increased at D4, D16, and at D12, D16 individually, indicating that water deficit has a delayed promoting effect on the expression levels of *PIF4* and *PIF7*.

The transcription of *PIF1*, *PIF3*, *PIF4* and *PIF7* were detected in strawberry. The expression levels of *PIF1* and *PIF3* in fruit were high, while the expression levels of *PIF4* and *PIF7* were relatively low. After two weeks of fruit setting, the expression level of *PIF1* and *PIF3* in strawberry fruit showed a downward trend, and the expression of *PIF4* and *PIF7* first decreased and then increased. The expression of *PIF1*, *PIF3*, *PIF4*, and *PIF7* was detected in mature fruit of citrus and the expression changes of *PIFs* were relatively stable, which may be due to the stability of endogenous metabolism in mature fruits. During the development of apple fruit, *PIF1*, *PIF1*-*like*, *PIF3*-*like*, *PIF4*-*like*, and *PIF7*-*like* all showed a gradually increasing trend. The expression of *PIF3* first increased and then decreased, indicating the expression changes of *PIFs* were not uniform and there may be some differences in the function of *PIFs* in different types of fruit (Figure S5).

## 4. Discussion

Light is an important environmental factor that regulates the development and coloration of grape berries. Therefore, the study of PIFs, as an important light signal regulator, is expected to be an interesting entry point for exploring the relationship between light and the appearance quality and intrinsic quality of grape berries. Due to the differences in chromosome location, amino acid sequence, and promoter sequences, the *PIFs* in grapes showed great differences in transcriptional levels and the potential functions.

### 4.1. PIFs Family and Their Evolutionary Analyses in Grapes

We identified a total of four *PIF* genes *PIF1*, *PIF3*, *PIF4*, and *PIF7* in the grape database, and they were distributed on four different chromosomes. By comparative alignment, many differences in the gene sequence and amino acid structure were detected. As gene exon-intron structures are typically conserved among homologous gene families [45], analysis of exon-intron structures will be helpful to reveal the evolutionary history of certain gene family [46].

From Figure 1, the *PIF1* gene structure and the number of exons and introns are relatively conserved in different species, but the gene length varies greatly, which is similar to the findings of [47]. On the other hand, the genes structure of *PIF3*, *PIF4*, and *PIF7* are not conserved in different plant species. Alterations in exon–intron structure within the coding region of a gene family might cause changes in their functions [48]. Compared with other fruit species, the gene lengths of *VvPIF3* and *VvPIF7* are longer. In grapes, *PIF1*, *PIF3*, and *PIF4* all contain 7 exons, while *PIF7* contains 6 exons. The second exon of each gene is the longest.

Phytochrome is an important functional protein that interacts with PIFs. The binding between phytochromes and PIFs depends on the correspondingly conserved sequences. Phytochrome-interacting factors have highly conserved APB motifs that are essential for PHYB binding [43]. AtPIF1 and AtPIF3 contain APA motifs respectively, which is also essential for the binding of PHYA [44,49]. Huq et al. [50] found that all AtPIFs have an APB motif and only PIF1, PIF3 have both an APB and an APA motif. In maize, there are at least seven putative PIFs with a conserved APB motif, of which two (ZmPIF3.1 and ZmPIF3.2) additionally also contain the APA motif, but only the interaction of ZmPIF3.1 and ZmPIF3.2 with ZmphyB1 has been proved now [51]. In grapes, the loss of the APB site in VvPIF1 may result in the inability of PHYB binding to this protein and affect the transmission of light signals. In terms of motifs in different species, PIF1, PIF3, and PIF4 are more conserved, and there are two specific motifs in PIF4 (Figure 4). Considering the existence of APB and APA [43,44,49,52], some conserved amino acids in motifs 10 and motifs 5 may be essential amino acids for maintaining the specific function of PIF4. PIF7 is less conserved in these species, and the function may be various in different plants.

Grapevines, like *Arabidopsis* and poplar, are dicotyledonous plants that diverged from monocotyledons about 130–240 million years ago [53]. Many dicotyledonous underwent whole genome triplication in recent million years, which made the duplications within PIF1, PIF7 and PIF8 clades in Solanum [54], grapevine genome has not undergone recent genome duplication, thus enabling the discovery of ancestral traits and features of the genetic organization of flowering plants. Combined with relative evolution time analysis, *PIF7* has an earlier evolutionary time, and *VvPIF7* may belong to the original *PIF* sequence. *VvPIF4* was formed later and may have certain plant specificity. Based on the results of collinear analysis, the functional analysis of *PIF1*, *PIF3*, and *PIF7* in model plants can contribute to the prediction of regulatory networks of corresponding *VvPIFs*.

### 4.2. Function Analysis of the PIFs

PIFs are important regulators of light signal transduction and each specific photoreaction can be regulated by several different PIFs [1,6,52]. PIF1 can inhibit the seeds germination. Both PIF1 and PIF3 promote hypocotyls negative gravitropism and inhibit the synthesis of chlorophyll. PIF4 and PIF5 can promote daily rhythmic growth of plants, and PIF7 can participate in enhancing shade avoidance of plants [1,52]. In recent years, researches on the synergies between PIF and other factors have gradually increased. Ni et al. [55] found that Photoregulatory Protein Kinases (PPKs) were collectively necessary for the normal light-induced phosphorylation and degradation of PIF3, and are critical components of a transcriptionally centered signaling hub that pleiotropically regulates plant growth and development in response to multiple signaling pathways. Kim et al. [56] identified the Repressor of Photosynthetic Genes1 *(RPGE1)* as a direct target of PIF1, which acted downstream of PIF1 in the endodermis to repress photosynthetic genes and regulate plastid development.

To clarify the metabolic network of PIFs in grapes, we performed promoter analysis and functional prediction of PIFs. The number of *cis*-elements related to photoreaction in the promoter of PIFs is the most abundant, which corresponds to the role of PIFs in the light response process.

*ZmPIF1* promoter region was rich in drought response (MBS) and ABRE-related elements, and that the expression level of *ZmPIF1* was significantly induced by drought and abscisic acid treatments [57]. In grapes, there is less *cis*-elements related to ABA response and no *cis*-elements related to drought response. The response of VvPIF1 to ABA and drought signals may not be as sensitive as ZmPIF1.

AtPIF3 is the main regulator promoting hypocotyls elongation in response to ethylene [9] and there is an ethylene response related element in its promoter. However, many ABA, instead of ethylene, responding related elements were found in the promoter of *VvPIF3*, which indicated that grapes may react differently from *Arabidopsis* in response to ABA and ethylene stimulation. PIF4 is the main regulator promoting hypocotyl elongation and early flowering response under high temperature [17,58]. There are only heat stress elements and no low-temperature response elements in the upstream of *VvPIF4*. *VvPIF4* was transcribed at low levels under the low temperature stress and might participate in the regulation of growth under high temperature. The transcription levels of *VvPIF4* and *VvPIF7* both increased under drought stress, but drought response elements were only detected in the promoter of *VvPIF7*, not in *VvPIF4*. *VvPIF7* and *VvPIF4* may respond to drought signals in different regulatory ways.

HY5 is a basic leucine zipper (bZIP) transcription factor that can form dynamic activation-suppression transcription module with PIFs, which directly act on the promoter *cis*-element G-box to cope with light and temperature changes. In regulating photosynthetic pigment synthesis genes, HY5 and PIFs do not operate alone, but with the circadian clock [59]. Through prediction of protein function, VvPIF1 may also interact with VvHY5 protein and participate in the regulation of related metabolic activities. GAI is a kind of DELLA protein, could function as negative modulators of PIF protein transcriptional regulatory activity by inhibiting their DNA-binding activities [60], The function of VvPIF3 and VvPIF4 may also be regulated by GAI protein.

*SlPIF1a* were identified in tomatoes. Upon induction of ripening, *PIF1a* transcript levels increased approximately fivefold in red ripe fruit compared to green samples [27]. In grapes, the expression pattern of *PIF1* in the skin of SM was similar to *SlPIF1a* (Figure 7), but that in the skin of SB was so different. These difference may be related to the accumulation of anthocyanins in SB skin, which were hardly synthesized in the skin of tomatoes and SM. Additionally, in the two grape cultivars, the transcription levels of *PIF4* and *PIF7* increased in the skin during the period before maturation while the expression of all *PIFs* generally showed a downward trend in the flesh, which indicated that the skin coloration didn’t affect or showed little effect on the expression of genes in the flesh at the transcriptional level. At the same time, after the shading treatment, the transcription level of PIFs in the skin and flesh of the two cultivars changed greatly, indicating that artificially changing the external light environment could directly or indirectly cause changes in the expression pattern of *PIFs* in the skin and flesh.

In *Arabidopsis*, the expression of *PIF4/5* was repressed during the beginning of the dark period and rose in the middle of the night to peak at dawn, whereas the transcript levels *PIF3*, and possibly *PIF1*, were relatively constant [61,62,63]. In grape leaves, for the shading treatment (Figure 8, red line from 2 h to 10 h), the expression levels of *PIF1*, *PIF3*, *PIF4*, *PIF7* all generally showed a firstly decreasing and then increasing trend, similar to the patterns of *AtPIF4* and *AtPIF5*. The specific expression pattern of *PIF1* and *PIF3* in grape, and the repression of *PIF3*, *PIF4* and *PIF7* expression at the end of dark treatment may be related to various factors. On the one hand, the test conditions of Arabidopsis and grapes were different. For example, the tinfoil was used to simulate the dark conditions of leaves in this study, but the whole plant was not treated darkly. On the other hand, Arabidopsis belongs to the herb while the grapes belong to the wood. Different plants have large differences in photomorphogenesis mechanisms, so the role of PIFs in plants, their expression patterns, and their sensitivity to light and darkness were also various.

Leaf shading experiments showed that *VvPIFs* were sensitive to light changes and shading treatment was beneficial to increase the expression level of *PIFs* though their reaction time was not the same. Recently, the function of PIFs in fruit trees has begun to be identified and functionally studied. Zhou et al. [47] identified the PIF1 in apple and found the PIF1 was not only involved in the germination of apple seeds and dormancy breaking of apple buds, but also inhibited the apple calli via PHY-mediated pathways. The function of various PIFs in grapes still needs further studies.

## 5. Conclusions

We identified PIF1, PIF3, PIF4, and PIF7 in the grapevine. These members are distributed on four different chromosomes with similar gene structures. *VvPIFs* (except *VvPIF4*) were located at the syntenic block with those from *A. thaliana*, *S. lycopersicum*, or *C. sinensis*. PIF3, PIF4, and PIF7 in grapes have conserved APB sequences. VvPIF1 has a conserved PIF1-specific APA sequence, while amino acid mutations occur in the specific APA sequences in VvPIF3. What’s more, two specific motifs are found in the PIF4 amino sequence. The photoreceptor-related elements in the *VvPIFs* promoter region are the most abundant. VvPIF1 and VvHY5, VvPIF3, VvPIF4 and VvGAI1 may interact with each other and participate in light signal transduction together.

The relative expression levels of *VvPIFs* varied in different organs at different developmental stages, *PIF4* was expressed at the highest level in most organs of grapevines. The transcriptional levels of *PIF4* and *PIF7* increased in the skin while the expression of all *PIFs* generally showed a downward trend in the flesh during the period before maturation. The transcription level of PIFs in grape leaves was sensitive to light/dark changes. Shading treatment was beneficial to enhance the transcription level of *VvPIFs*, but the effect is time-controlled on *VvPIF3* and *VvPIF4*. These findings could lay the theoretical foundation for the function study of PIFs and the further construction of grape light regulation networks.

## Figures and Tables

**Table 1:** Characteristics of Phytochrome-Interacting Factors (*PIFs*) genes.

Name	Locus Id	Genomic DNA Size (bp)	Number of Amino Acids	Predicted Mw (kDa)	Theoretical pI	Chromosome Location	Functional Domains (Start-End, bp)
*PIF1*	LOC100264869	5238	516	56.32	5.32	7	308-357/bHLH
*PIF3*	LOC100247781	5463	709	75.861	6.3	14	462-511/bHLH
*PIF4*	LOC100262490	3798	531	57.765	7.3	12	338-387/bHLH
*PIF7*	LOC100262138	3617	423	46.631	8.94	17	223-272/bHLH

Note: bHLH: basic helix–loop–helix.

**Table 2:** Coding region nucleotide (upper portion of matrix) and amino acid (bottom portion of matrix) sequence pairwise comparisons (% similarity) between grape *PIF* genes.

	VvPIF1	VvPIF3	VvPIF4	VvPIF7
**VvPIF1**	-	48/29	55/19	51/24
**VvPIF3**	26/34/39	-	50/29	44/41
**VvPIF4**	33/42/24	27/38/28	-	49/26
**VvPIF7**	28/41/20	22/31/41	25/35/34	-

Note: This table is divided into two parts, the upper part (upper right corner) representing the nucleotide comparison between different *PIFs*, and the lower part (lower left corner) representing the comparison of protein sequences between different PIFs. The numbers represent Identities/Positives/Gaps (lower left corner) ratio, and Identities/Gaps (upper right corner) ratio respectively.

## Supplementary Materials

The following are available online at http://www.mdpi.com/2073-4425/9/9/451/s1, Table S1. Synteny regions of FIFs genes between grape and *Arabidopsis thaliana*, *Solanum lycopersicum*, *Citrus sinensis*, Figure S1. The amino acid sequence of PIFs motifs in MEME analysis, Figure S2. The interaction protein prediction of VvPIFs, Figure S3. Expression patterns of the grape PIFs family in different organs of ‘Corvina’, Figure S4. Expression patterns of the grape PIFs family in the leaves of ‘Cabernet Sauvignon’ under long or short term abiotic stress, Figure S5. Expression patterns of the PIFs family in the fruits of strawberry, citrus, and apple, Table S2. List of primer sequences used in qPCR analysis, Table S3. Prediction functional partners of PIFs and their annotation.
