# Supplementary material for: Genome-Wide Identification of PIFs in Grapes (Vitis vinifera L.) and Their Transcriptional Analysis under Lighting/Shading Conditions
Source: Genes (Basel). 2018 Sep 7;9(9):451. doi: 10.3390/genes9090451 (PMC6162725; doi:10.3390/genes9090451)
Supplement: Supplementary file 1 [file genes-09-00451-s001.pdf]

Table S1 Synteny regions of FIFs genes between grape and *Arabidopsis,Solanum lycopersicum.Citrus sinensis*

**Gene1**	**Gene ID**	**Chr**	**Start**	**End**	**Gene2**	**Gene ID**	**Chr**	**Start**	**End**	**E-value**
SlPIF3	XP_010313958.1	Slchr1	91122473	91127758	AtPIF3	AT1G09530.1	Atchr1	3076582	3079539	4.00E-68
SlPIF3	XP_010313958.1	Slchr1	91122473	91127758	CsPIF3	XP_006480339.1	Cschr6	1164034	1170166	5.00E-162
SlUNE10	XP_004236485.1	Slchr3	65211815	65214366	AtPIF7	AT5G61270.2	Atchr5	24638773	24640401	4.00E-27
SlUNE10	XP_004236485.1	Slchr3	65211815	65214366	CsPIF7	XP_006477612.1	Cschr5	8510879	8514543	3.00E-105
SlPIF1	XP_004247109.1	Slchr9	60928082	60939349	AtPIF4	AT2G20180.1	Atchr2	8704024	8706892	2.00E-73
SlPIF1	XP_004247109.1	Slchr9	60928082	60939349	CsPIF1	XP_006480179.1	Cschr5	35349068	35355417	3.00E-158
VvPIF3	XP_010659764.1	Vvchr14	262238	267700	AtPIF3	AT1G09530.1	Atchr1	3076582	3079539	7.00E-84
VvPIF3	XP_010659764.1	Vvchr14	262238	267700	CsPIF3	XP_006480339.1	Cschr6	1164034	1170166	0.00E+00
VvPIF7	XP_002284441.1	Vvchr17	7609528	7613144	CsPIF7	XP_006477612.1	Cschr5	8510879	8514543	2.00E-144
VvPIF7	XP_002284441.1	Vvchr17	7609528	7613144	SlUNE10	XP_004236485.1	Slchr3	65211815	65214366	1.00E-108
VvPIF1	XP_002263397.3	Vvchr7	9032951	9038188	SlPIF1	XP_004247109.1	Slchr9	60928082	60939349	9.00E-159

Figure S1. The amino acid sequence of PIFs motifs in MEME analysis. The number of each sequence corresponds to the motifs number.

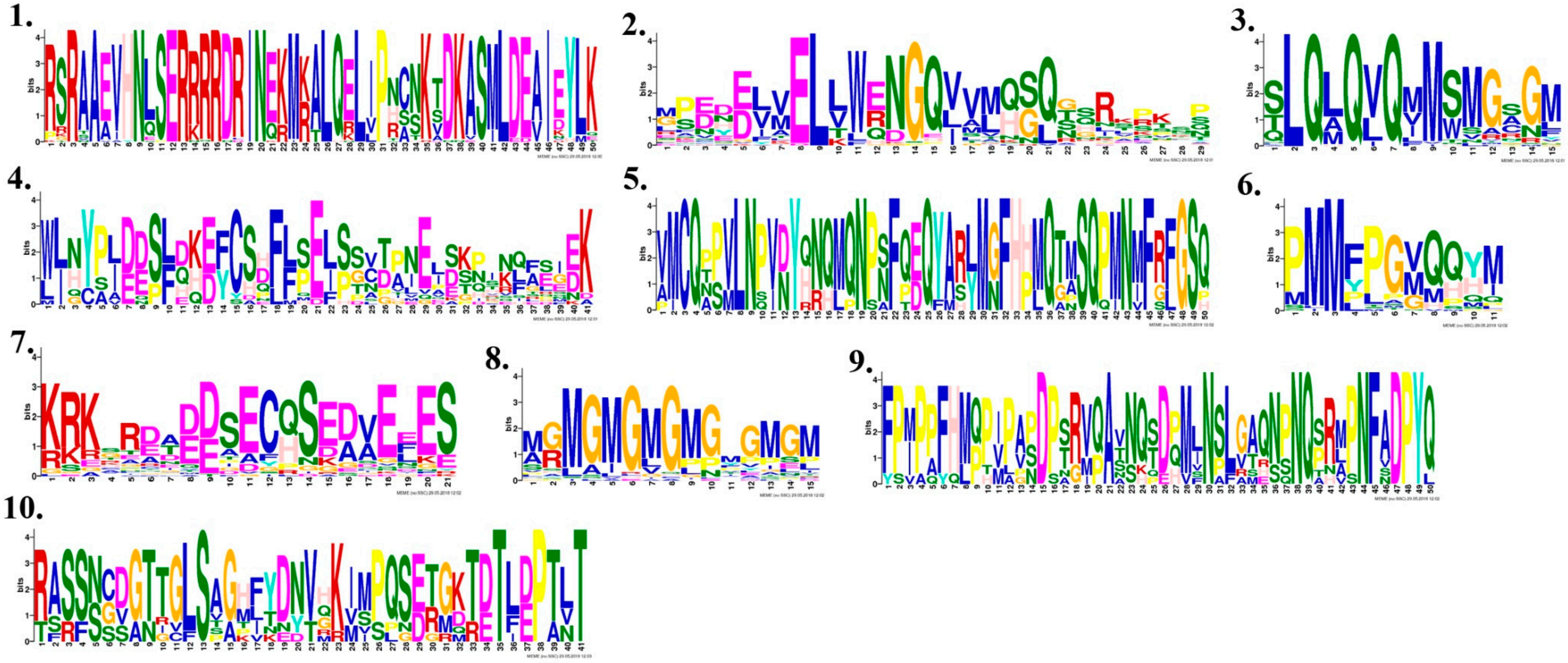

Figure S2. The interaction protein prediction of VvPIFs. The red ball in A, B, C represents VvPIF1, VvPIF3, VvPIF4, respectively,

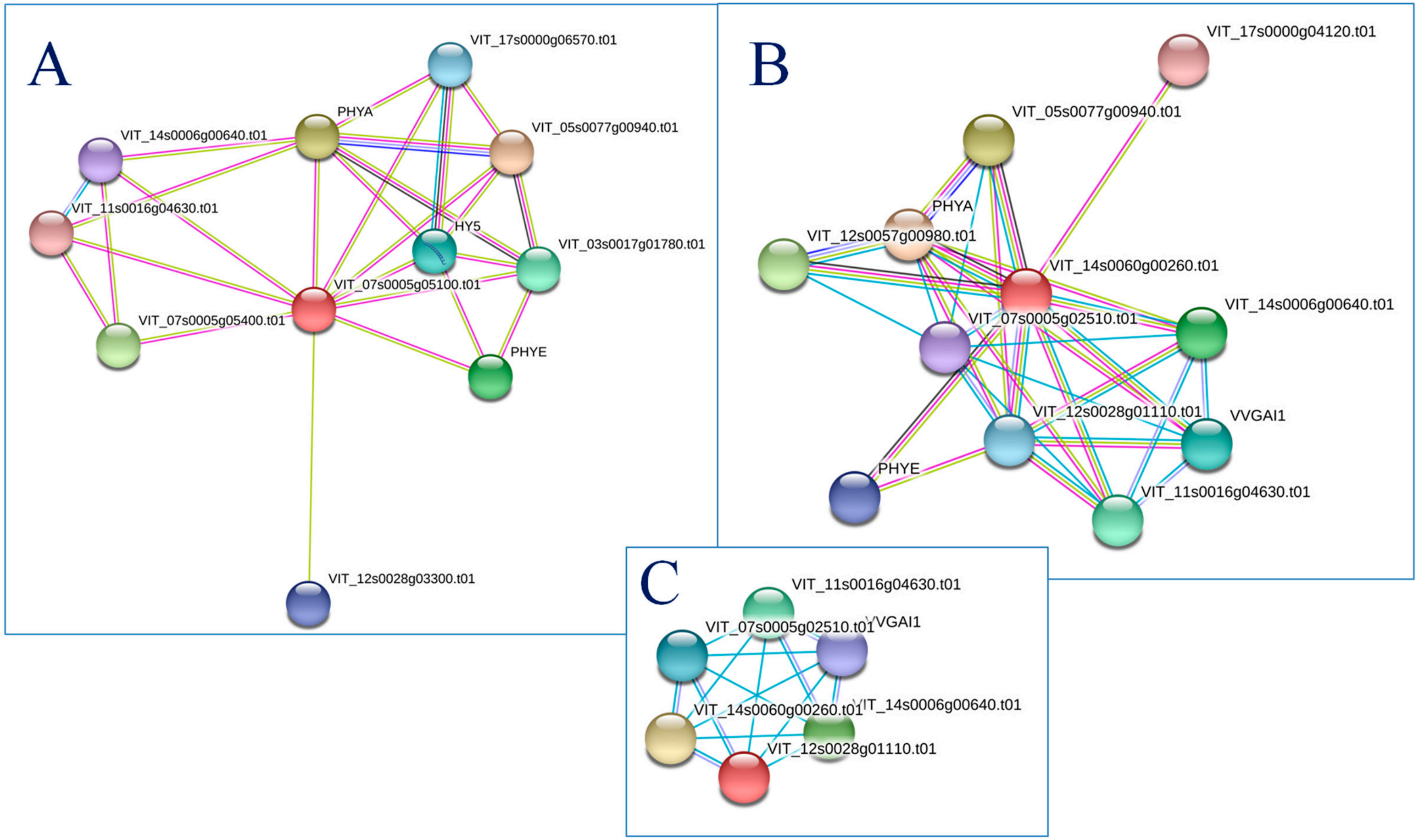

Figure S3. Expression patterns of the grape PIFs family in different organs of ‘Corvina’. The colour scale in the heat map represent expression values: red represent high transcript abundance while green represent low level of transcript abundance. The expression value was from GSE36128 in NCBI. The sampling period corresponding to the word is as follows: FS: fruit set, PFS: post-fruit set, V: veraison, MR: mid-ripening, R: ripening, S:senescencing, B: burst, AB: after-burst, L:latent, Bud-W: winter bud, Y: young, WD: well developed, FB: flowering begins, F: flowering, G:green, Stem-W: woody stem.

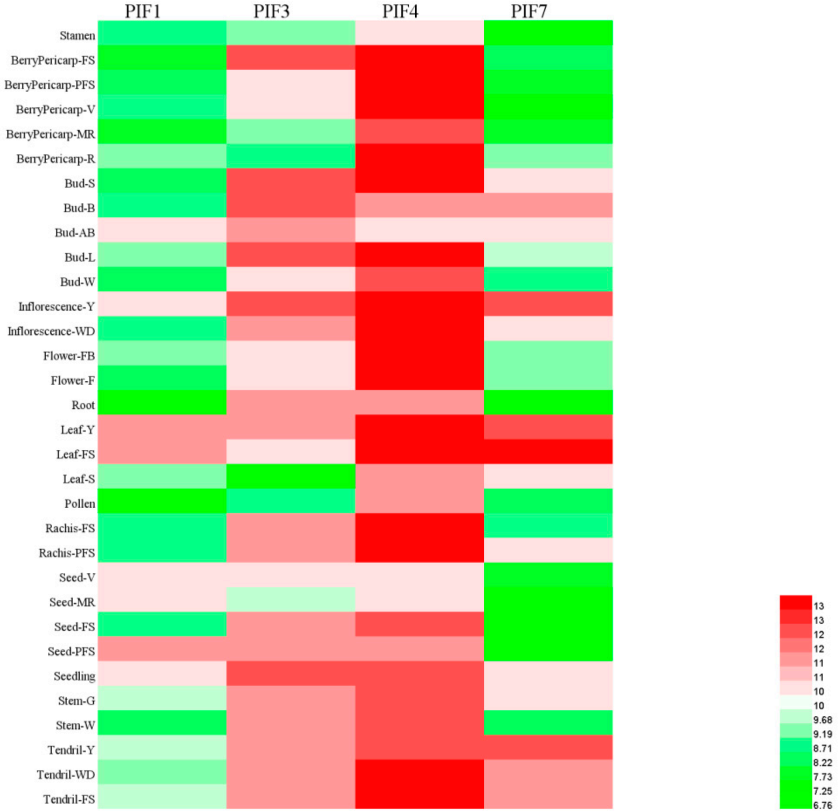

Figure S4. Expression patterns of the grape PIFs family in the leaves of 'Cabernet Sauvignon' under long or short term abiotic stress. A,C represent the changes of PIF4, B,D represent the changes of PIF4. The colour scale up the heat map represent expression values: red represent high transcript abundance while green represent low level of transcript abundance. The expression value was from GSE31594 and GSE31677 in NCBI.

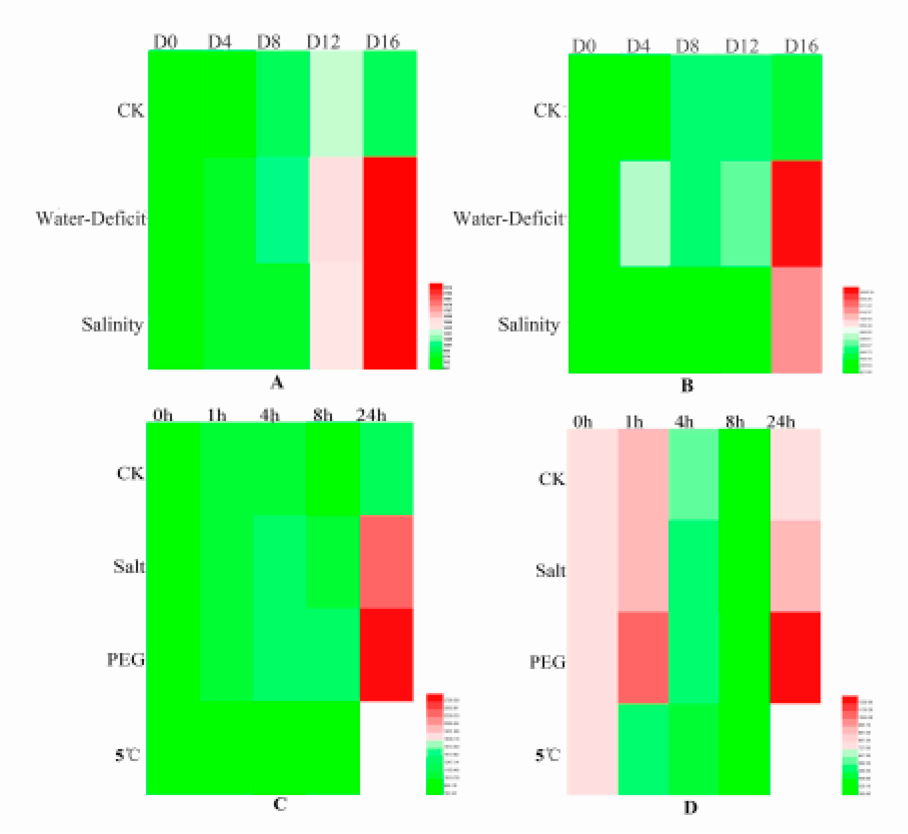

Figure S5. Expression patterns of the PIFs family in the fruits of strawberry, citrus, and apple. The colour scale up the heat map represent expression values: red represent high transcript abundance while green represent low level of transcript abundance. The expression value was from GSE85572, GSE69432, and GSE64079.

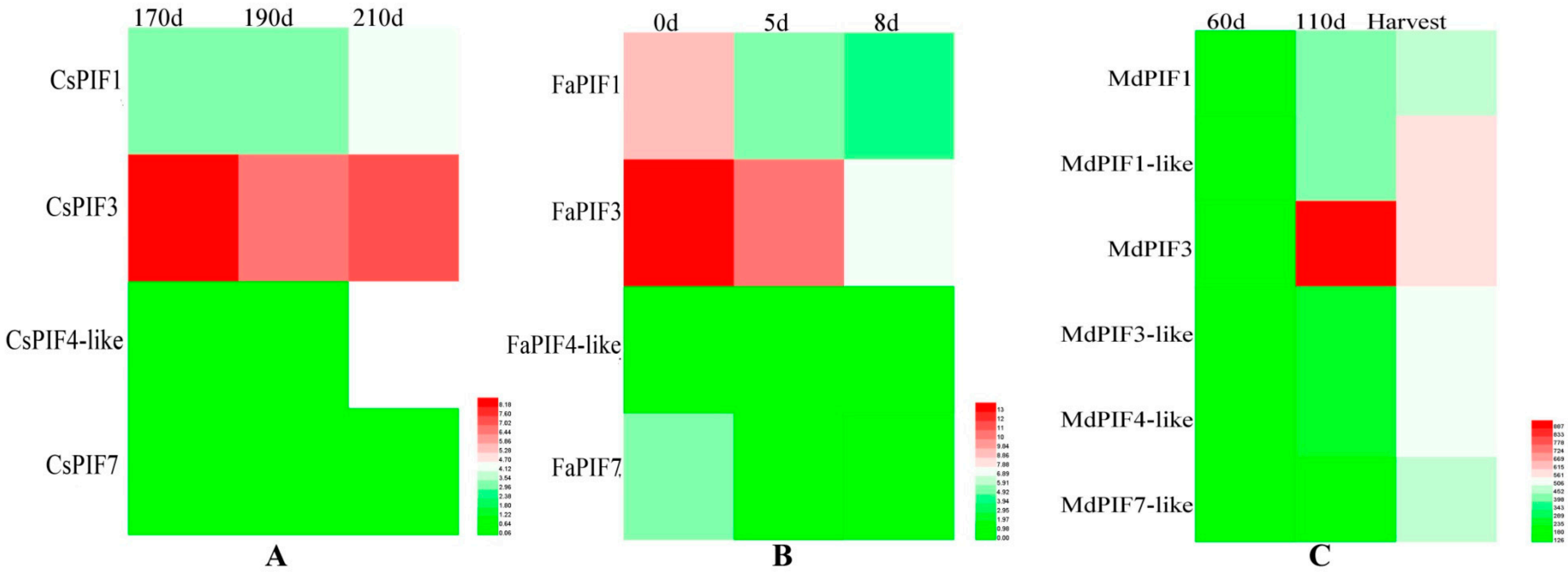

Table S2. List of primer sequences used in qPCR analysis.

Gene	Sequence of forward (5'-3') and reverse primers (5'-3')
PIF1	F: 5'- ACATGGCTGCACCTGATTCATCTA-3'
	R: 5'- GCTGGTACTTGGCTTGCTCGTA-3'
PIF3	F: 5'- ATGGGAAAGTTCGGGTCAATGGAT-3'
	R: 5'- CAGTGACACCGGATAATTCTTGCA-3'
PIF4	F: 5'- CGGTCTTGCTCCAAATGAGTCTAA-3'
	R: 5'- GGGCGGCAATTCCATGAACAAA-3'
PIF7	F: 5'- TTTGTCCCTCCTCCCTTTGTAGTG-3'
	R: 5'-TGCTTGTTGGTTGTGCCTTGTG-3'
UBIQUITIN1	F: 5'-TCTGAGGCTTCGTGGTGGTA-3'
	R: 5'-AGGCGTGCATAACATTTGCG-3'
EF1γ	F: 5'-CAAGAGAAACCATCCCTAGCTG-3'
	R: 5'-TCAATCTGTCTAGGAAAGGAAG-3'

Table S3. Prediction functional partners of PIFs and their annotation.

Protein	Predicted Functional Partners:	Annotation	Scores	KEGG Pathways
PIF1	VIT_05s0077g00940.t01	Putative uncharacterized protein; Regulatory photoreceptor which exists in two forms that are reversibly interconvertible by light- the Pr form that absorbs maximally in the red region of the spectrum and the Pfr form that absorbs maximally in the far-red region (1129 aa)	0.819	Circadian rhythm - plant(pathway ID4712)
	PHYA	Phytochrome; Regulatory photoreceptor which exists in two forms that are reversibly interconvertible by light- the Pr form that absorbs maximally in the red region of the spectrum and the Pfr form that absorbs maximally in the far-red region (1124 aa)	0.808	
	VIT_07s0005g05400.t01	Putative uncharacterized protein (733 aa)	0.795	
	PHYE	Putative uncharacterized protein (1054 aa)	0.761	
	VIT_03s0017g01780.t01	Putative uncharacterized protein (847 aa)	0.652	
	HY5	Putative uncharacterized protein (169 aa)	0.525	
	VIT_17s0000g06570.t01	Putative uncharacterized protein (551 aa)	0.493	
	VIT_12s0028g03300.t01	Putative uncharacterized protein (360 aa)	0.487	
	VIT_14s0006g00640.t01	Putative uncharacterized protein (569 aa)	0.456	
	VIT_11s0016g04630.t01	Putative uncharacterized protein (532 aa)	0.456	
PIF3	PHYA	Phytochrome; Regulatory photoreceptor which exists in two forms that are reversibly interconvertible by light- the Pr form that absorbs maximally in the red region of the spectrum and the Pfr form that absorbs maximally in the far-red region (1124 aa)	0.965	Circadian rhythm - plant(pathway ID4712) and Plant hormone signal transduction(pathway ID4075)
	VIT_05s0077g00940.t01	Putative uncharacterized protein; Regulatory photoreceptor which exists in two forms that are reversibly interconvertible by light- the Pr form that absorbs maximally in the red region of the spectrum and the	0.964	

		Pfr form that absorbs maximally in the far-red region (1129 aa)		
	VIT_12s0057g00980.t01	Putative uncharacterized protein; Regulatory photoreceptor which exists in two forms that are reversibly interconvertible by light- the Pr form that absorbs maximally in the red region of the spectrum and the Pfr form that absorbs maximally in the far-red region (1118 aa)	0.912	
	VIT_14s0006g00640.t01	Putative uncharacterized protein (569 aa)	0.885	
	VIT_11s0016g04630.t01	Putative uncharacterized protein (532 aa)	0.885	
	VVGAI1	DELLA protein GAI1 (590 aa)	0.885	
	VIT_12s0028g01110.t01	Putative uncharacterized protein (558 aa)	0.86	
	PHYE	Putative uncharacterized protein (1054 aa)	0.823	
	VIT_07s0005g02510.t01	Putative uncharacterized protein (200 aa)	0.8	
	VIT_17s0000g04120.t01	Putative uncharacterized protein (697 aa)	0.759	
PIF4	VIT_14s0060g00260.t01	Putative uncharacterized protein (709 aa)	0.8	Circadian rhythm - plant(pathway ID4712) and Plant hormone signal transduction(pathway ID4075)
	VIT_14s0006g00640.t01	Putative uncharacterized protein (569 aa)	0.8	
	VIT_11s0016g04630.t01	Putative uncharacterized protein (532 aa)	0.8	
	VIT_07s0005g02510.t01	Putative uncharacterized protein (200 aa)	0.8	
	VVGAI1	DELLA protein GAI1 (590 aa)	0.8	
